# Adaptive radiation of gobies in the interstitial habitats of gravel beaches accompanied by body elongation and excessive vertebral segmentation

**DOI:** 10.1186/1471-2148-9-145

**Published:** 2009-06-28

**Authors:** Tomohiko Yamada, Tomoshige Sugiyama, Nana Tamaki, Atsushi Kawakita, Makoto Kato

**Affiliations:** 1 Graduate School of Human and Environmental Studies, Kyoto University, Yoshida-Nihonmatsu-cho, Sakyo, Kyoto 606-8501, Japan

## Abstract

**Background:**

The seacoasts of the Japanese Arc are fringed by many gravel beaches owing to active tectonic uplift and intense denudation caused by heavy rainfall. These gravel beaches are inhabited by gobies of the genus *Luciogobius *that burrow into the gravel sediment and live interstitially. Although their habitat and morphology (*e. g*., reduced fins, elongated, scale-less body, and highly segmented vertebral column) are highly unusual among fishes, little is known on how their morphological evolution has facilitated the colonization of interstitial habitats and promoted extensive diversification. We conducted thorough sampling of *Luciogobius *and related species throughout Japan, and performed molecular phylogenetic analysis to explore the patterns of morphological evolution associated with gravel beach colonization.

**Results:**

An analysis of the mitochondrial *cytochrome b *gene suggested a remarkable diversity of previously unrecognized species. The species-level phylogeny based on six protein-coding nuclear genes clearly indicated that interstitial species cluster into two distinct clades, and that transitions from benthic or demersal habits to interstitial habits are strongly correlated with an increase in vertebral number. Colonization of gravel beach habitats is estimated to have occurred ca. 10 Ma, which coincides with the period of active orogenesis of the Japanese landmass. Different species of interstitial *Luciogobius *inhabit sediments with different granulometric properties, suggesting that microhabitat partitioning has been an important mechanism facilitating speciation in these fishes.

**Conclusion:**

This is the first study to document the adaptation to interstitial habitats by a vertebrate. Body elongation and excessive vertebral segmentation had been the key aspects enhancing body flexibility and fishes' ability to burrow into the gravel sediment. The rich diversity of coastal gravel habitats of the Japanese Arc has likely promoted the adaptive radiation of these unique gravel-dwelling fishes.

## Background

**Figure 1:**
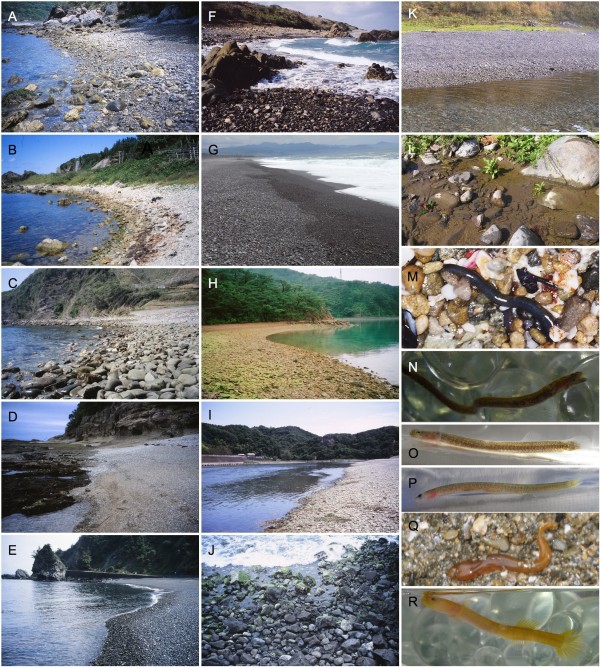
***Luciogobius *fishes and their habitats**. **A**. Gravel beach at Nagashima inhabited by *L. grandis, L. platycephalus*, *L. parvulus *and *L. elongatus*; **B**. Gravel beach at Kitaebisu inhabited by *L. grandis*; **C**. Boulder beach at Tsushima inhabited by *L. grandis *and *L. parvulus*; **D**. Gravel beach at Shirahama inhabited by *L. grandis *and *L. elongatus*; **E**. Gravel beach at Kurogahama inhabited by *L. grandis *and *L. elongatus*; **F**. Coarse gravel beach at Yaku Island inhabited by *L. grandis*; **G**. Coarse gravel beach at Shichiri-Mihama inhabited by *L*. sp.; **H**. Calm gravel beach at Chinoura inhabited by *L. elongatus*; **I**. Tonda River estuary inhabited by *L. pallidus*; **J**. Rocky shore at Hachijo Island inhabited by *L. elongatus*; **K**. Gravel beach at Tappi inhabited by *L. grandis, L. elongatus, L. platycephalus *and *L*. sp.; **L**. Ochikabake River estuary inhabited by *L. guttatus*; **M**. *L. grandis *found in intertidal gravel sediment at Shirahama; **N-O**. *L. elongatus*; **P**. *L. adapel*; **Q**. *L. pallidus*; and **R**. Gen. sp. 1.

**Figure 2:**
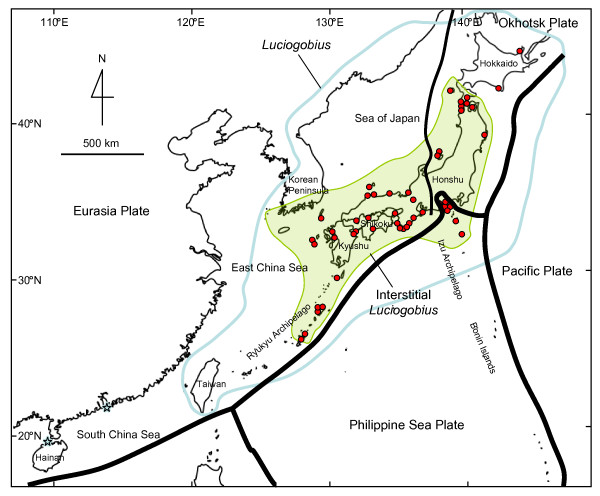
**Map of East Asia showing *Luciogobius *distribution and sampling localities**. Positions of the tectonic plates and their margins are also shown. Distribution range of *Luciogobius *is indicated in blue, and that of the interstitial species are indicated in green. Stars indicate outlying distributions of *L. brevipterus *and *L. martellii *in Hainan Island and Hong Kong, respectively.

Fishes have undergone remarkable adaptations to utilize various coastal environments, including sandy beaches, mud flats, rocky shores, coral reefs, and mangroves [1]. Another, less exploited, coastal habitat is gravel beaches, where the shore sediment consists mainly of gravels [2-4]. Gravel beaches harbor diverse interstitial invertebrates [5], but the perpetual stirring of gravel by the waves usually make them unsuitable as habitat for vertebrates. However, gobies of the East Asian genus *Luciogobius *have elongated, flexible bodies and are adapted to inhabit the dynamic sediment of gravel beaches (Figure 1) [6-11]. The genus is characterized by the loss of the first dorsal fin, an elongated, scale-less body, and a drastic increase in the number of vertebrae [12,13], which are putative morphological adaptations to the interstitial lifestyle. So far, 11 *Luciogobius *species have been described from the Japanese Arc, Taiwan, Hainan Island, Hong Kong, and the southernmost seacoasts of the Russian Far East and Korean Peninsula (Figure 2). Seven of these species are endemic to Japan [13-15]. Five of the 11 species are strongly associated with well-sorted gravel beach sediment and are strictly interstitial [11,16-19] (Table 1); they reside within the gravel throughout their life after a pelagic larval stage and prey on interstitial invertebrates [16-19].

**Table 1:** List of currently described *Luciogobius *species and additional taxa sampled in this study.

Genus Species	Habitat	Vertebra number	Organs lost
*Luciogobius*			
*elongatus*	interstitial, intertidal gravel beach	38–44	scale; first dorsal fin
*adapel*	interstitial, gravel bed (20–40 m b.s.l.)	50	scale; first and second dorsal, anal, pelvic
*grandis*	interstitial, intertidal gravel beach	40–42	scale; first dorsal fin
*platycephalus*	interstitial, intertidal gravel beach	41–42	scale; first dorsal fin
*parvulus*	interstitial, intertidal gravel beach	41–43	scale; first dorsal and pelvic fins
*guttatus*	under stone in estuary or protected rocky shore	35–39	scale; first dorsal fin
*pallidus*	mudshrimp burrow, underground water	37	scale; eye; first dorsal fin; chromatophore
*Albus*^†^	anchialine cave, underground water	30–31	scale; eye; first dorsal fin; chromatophore
*dormitoris*^†^	estuary (habitat little known)	36	scale; eye; first dorsal fin; chromatophore
*brevipterus*^†^	habitat information unavailable	35	scale; first dorsal fin
*martellii*^†^	habitat information unavailable	33	scale; first dorsal fin
sp.	interstitial, intertidal gravel beach	45–46	scale; first dorsal fin
*Inu*			
*koma*	intertidal rocky shore	31–32	scale reduced; first dorsal fin
*saikaiensis*	intertidal rocky shore	32–33	scale; first dorsal fin
Gen.			
sp. 1	intertidal rocky shore, underground water	34–35	scale; first dorsal fin
sp. 2	intertidal rocky shore	37	scale; first dorsal fin
*Clariger*			
*cosmurus*	subtidal rocky shore	33	scale; first dorsal fin
*Exilis*	subtidal rocky shore	33	scale; first dorsal fin
*Astrabe*			
*flavimaculata*	subtidal rocky shore	30	first dorsal fin reduced
*Chaenogobius*			
*gulosus*	subtidal rocky shore	33	
*Gymnogobius*			
*isaza*	freshwater lake	33	
*urotaenia*	freshwater lake, river	32	
*Typhlogobius*			
*californiensis*	intertidal rocky shore, mudshrimp burrow	30–32	eye, chromatophore
*Eutaeniichthys*			
*gilli*	estuary	39	
*Leucopsarion*			
*petersi*	anadromous, migratory (pelagic in the sea)	33	first dorsal fin

^†^Taxa not sampled in this study.

Among East Asian seacoasts, those surrounding the Japanese Arc are particularly rich in gravel beaches [4]. This is because the Japanese Arc is located on compressional plate margins and has experienced active orogenesis since the Middle Miocene (ca. 15 Ma) [20-22]. The resultant steep mountain ranges and abundant rainfall produced gravely riverbeds and gave rise to many gravel beaches along the seacoasts of Japan. Consequently, the diversity and endemicity of *Luciogobius *gobies may have been shaped by the richness of gravel beach habitats along the coastline of the Japanese Arc. However, the actual diversity and patterns of morphological evolution associated with the interstitial habits of the genus remain largely unexplored. Examination of the evolutionary history of these unique gobies could produce a model for understanding the processes and mechanisms of adaptation by vertebrates to dynamic interstitial habitats.

In this study, we conducted a molecular phylogenetic analysis based on extensive sampling of *Luciogobius *gobies and allied genera from the seacoasts of Japan (Figure 2), to clarify their diversity and the evolutionary history of their interstitial habits. We focused on the correlated evolution of interstitial habits and vertebra number, because, despite being among the smallest gobies, *Luciogobius *species have vertebra numbers (*i. e*., 35–50) that far exceed those typically found in other Gobiidae (*i. e*., 26) [12]. The elongated body and highly segmented vertebral column likely enhance flexibility and increase the fishes' ability to burrow into the gravel sediment. The obtained phylogenetic tree was then used to estimate the timing of the evolution of interstitial habitat use, which is interpreted in light of the geological history of the Japanese Arc. We also compared the granulometric properties of gravels inhabited by different species of interstitial *Luciogobius *to determine if there is niche partitioning among species, which may have contributed to the evolution and maintenance of the present diversity of *Luciogobius*.

## Results

### Phylogenetic analysis

**Figure 3:**
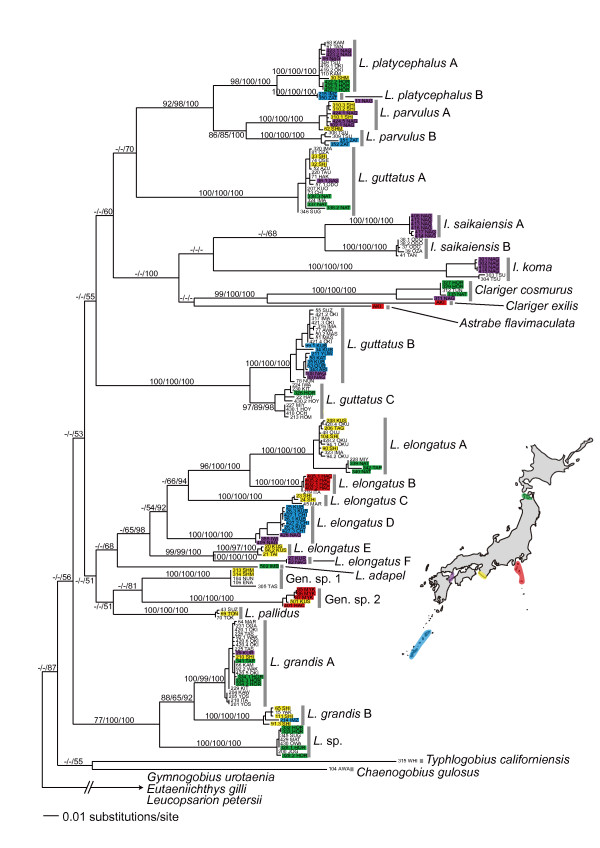
**Maximum likelihood phylogeny based on 608 bp of the mitochondrial *Cyt b *gene**. Numbers above branches indicate maximum parsimony and maximum likelihood bootstrap values, followed by Bayesian posterior probabilities. Areas with particularly high number of *Luciogobius *species are color-shaded on the map of Japan; specimens from each of these areas are marked with the corresponding color.

**Figure 4:**
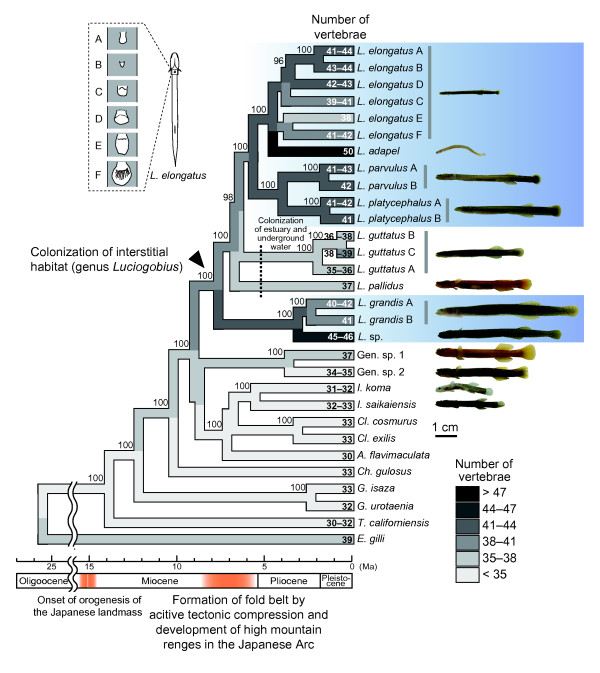
**Chronogram based on the maximum likelihood phylogeny of the combined *Mll*, *Myh6*, *Ptr*, *Rag1*, *Rag2*, and *Ryr3 *genes**. Numbers above nodes indicate Bayesian posterior probabilities, and those at terminal branches indicate vertebra number of each species. The ancestral state reconstructions of vertebra number are also shown. Interstitial species are indicated by the blue background. Illustrations of pelvic fin morphologies are provided for the six genetically distinct groups of *L. elongatus*.

To determine whether *Luciogobius *species contain any previously unrecognized taxa, we first sequenced the mitochondrial cytochrome *b *(*Cyt b*) gene for all sampled gobies (190 individuals). Phylogenetic analysis of the *Cyt b *data suggested that six of the seven sampled *Luciogobius *species each contain genetically divergent clades that are separated by 5–12% uncorrected pairwise sequence difference (Figure 3). *Luciogobius elongatus *had the highest number of clades (six), while *L. guttatus *was separated into three clades, and the others were each grouped into two clades. The divergent clades within each species formed a monophyletic group, except for *L. guttatus*, which was not recovered as monophyletic in the *Cyt b *tree (but see results of the combined nuclear analysis). Although most of the intraspecific clades did not overlap in their distributions, those within *L. guttatus*, *L. elongatus*, and *L. grandis *co-occurred in some locations (Figure 3), suggesting that the observed genetic differentiation is not simply due to geographic isolation. While detailed morphological examinations are currently underway, at least some pairs of genetically differentiated clades have distinct morphological differences; the six clades within *L. elongatus *can be distinguished from each other by vertebra number and the morphology of the pelvic fin (Figure 4). Therefore, we treat each of the well-defined clades as distinct species in the following analyses (see Discussion for further arguments on species status). In addition to the above previously unrecognized taxa, gobies that could not be assigned morphologically to presently described species were grouped into three well-supported clades (*L*. sp. and Gen. sp. 1 and 2 in Figure 3).

Because the *Cyt b *gene evolves too rapidly and is therefore not sufficiently informative for examining species-level relationships, we also sequenced six protein-coding nuclear genes to recover phylogenetic relationships among *Luciogobius *species and related gobies. One representative individual was chosen from each species arbitrarily, including the *Luciogobius *taxa genetically revealed by the *Cyt b *data, and sequenced for the following six genes: recombination activating genes 1 and 2 (*Rag1 *and *Rag2*); myeloid/lymphoid or mixed-lineage leukaemia (*Mll*); protease III (*Ptr*); ryanodine receptor 3 (*Ryr3*); and myosin, heavy chain 6, cardiac muscle, alpha (*Myh6*). Phylogenetic analysis based on the combined nuclear gene dataset produced a well-resolved phylogeny with high nodal support values (Figure 4 and Additional file 1). The genus *Luciogobius *is monophyletic and does not include *I. koma *and *I. saikaiensis*, which are treated as species of *Luciogobius *in most recent classifications [13]. The interstitial species (Table 1) separated into two distinct clades within *Luciogobius*. The three genetically divergent clades within *L. guttatus*, which were not recovered as monophyletic in the *Cyt b *tree, grouped into a single, well-supported clade.

### Evolution of vertebra number

The vertebra numbers of *Luciogobius *species are among the highest of all gobies known [12]. The increase in the number of vertebrae likely enhances body flexibility, and the transition to interstitial habitats may be associated with increases in vertebra number. To test this hypothesis, we counted the number of vertebrae for each taxa identified from the *Cyt b *phylogeny (Additional file 2). The number of vertebrae varied little within species, but greatly across the sampled goby species, ranging from 30 in *Astrabe flavimaculata *to 50 in *L. adapel*. Using the above species-level phylogeny, we reconstructed ancestral character states of vertebra number (Figure 4), which shows that clades of interstitial species have markedly higher numbers of vertebrae. Regression using independent contrasts [23] indicated that the probability of transition to interstitial habitats is strongly correlated with an increase in vertebra number (*r *= 0.605, *P *= 0.00064).

### Estimation of divergence time

To determine the timing of the origin of interstitial habit in *Luciogobius*, we estimated divergence times based on the combined nuclear gene phylogeny. Fossil data are commonly used to calibrate divergence times, but fossils belonging to the gobiid group under study are not currently available. Instead, we calibrated the tree using (1) the split between *Gymnogobius isaza *and *G. urotaenia*, which was previously estimated at 2.3 Ma based on geological calibration [24] and (2) the divergence rate of the *Cyt b *gene (approximately 2.7% per My in gobiid taxa [24]). Since a likelihood ratio test rejected a strict molecular clock for the combined nuclear gene dataset (*P *< 0.001), we employed the penalized-likelihood relaxed clock approach using the program r8s [25].

Because a reliable maximum prior for the age of the root node is unavailable, we first obtained relative node ages by fixing the age of the root node at one arbitrary time unit without any internal constraints. The obtained ultrametric tree was then calibrated by fixing the *G. isaza*-*G. urotaenia *split at 2.3 Ma, which suggested that the most recent common ancestor (MRCA) of interstitial *Luciogobius *species dates back to 7.67 Ma (95% credibility interval, 5.52–12.27 Ma) (Figure 4). We also estimated ages of all the internal nodes individually based on average pairwise ML distances across each node and the 2.7% *Cyt b *divergence rate. These estimates were then individually used as calibration points to infer the age of the MRCA of interstitial *Luciogobius *on the above ultrametric tree. Results of this analysis converged on a similar range of estimates for the age of interstitial *Luciogobius*, as nodes used to calibrate the tree became deeper on the phylogeny (Additional file 3). Although older estimates were obtained when calibrated with recent nodes, these outlying values are likely due to stochastic error associated with using shallowly placed nodes for deep extrapolations [26,27].

In addition to these analyses, we also estimated divergence times by obtaining the age of the root node with an iterative approach [28]. This was done by adjusting the age of the root node until average rate of *Cyt b *substitution (calculated using r8s) equalled the previously inferred rate of 0.0135, which is half the 2.7% divergence rate [24]. This method resulted in estimates of 27.7 Ma for the root node (Additional file 4) and 8.11 Ma (95% credibility interval, 6.89–9.58 Ma) for the MRCA of the interstitial *Luciogobius*, which is comparable to the values obtained using the above two approaches.

### Habitat assessment

**Figure 5:**
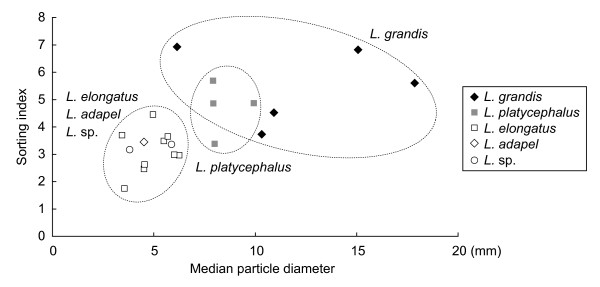
**Plots of median particle diameter and sorting index of gravel samples collected for five interstitial *Luciogobius *species**.

To determine whether different species of interstitial *Luciogobius *prefer habitats with different gravel sizes, we quantified the median gravel size and sorting index of the sediment [29]; these are granulometric measures used to describe the properties of sediments. The sorting index describes how well the gravels are sorted by size and ranges between 0 and 1, with better-sorted gravels having lower sorting indices. Using the median diameter and sorting index as descriptors of gravels, the five interstitial *Luciogobius *species analyzed were largely grouped into three habitat types (Figure 5 and Additional file 5): *L. grandis *inhabiting relatively large, less sorted gravel sediments, *L. platycephalus *inhabiting intermediate-sized gravel sediments, and *L. elongatus*, *L. adapel*, and *L*. sp. inhabiting small, well-sorted gravel sediments. There were significant differences among *L. grandis*, *L. platycephalus*, and *L. elongatus *in both median gravel size (ANOVA, *F*_2,17 _= 13.36, *P *< 0.001) and sorting index (*F*_2,17 _= 9.59, *P *= 0.002), suggesting that they utilize gravel habitats with broadly different granulometric properties. Data on *L. adapel *and *L*. sp. were not included in the test due to insufficient sample sizes.

Although the six species within the *L. elongatus *group (sp. A-F) inhabited sediments with similar granulometric parameters, their microhabitats differed from one another with respect to other aspects of the beach. Species A inhabited intertidal gravel banks on turbulent coasts, whereas sp. B was found in the intertidal sediments of lava gravel on rocky shores, sp. C in intertidal gravel sediments on bedrock, sp. D in the upper intertidal zone of protected gravel beaches in inner bays, sp. E in infiltrations of fresh water running through gravel sediments on boulder beaches, and sp. F in the lower layer of intertidal gravel sediments on boulder beaches. Although further, detailed habitat assessment is necessary, these species with their different vertebra numbers and pelvic fin morphologies, prefer specific interstitial microhabitats characterized by wave action, tidal level, and the presence or absence of boulders, rocks, bedrock, and inflow of infiltrated freshwater.

## Discussion

### Diversity and phylogeny of *Luciogobius*

Overall, our discovery of numerous previously unrecognized species within *Luciogobius *greatly advanced our understanding of the diversity in the genus. Different taxa within currently recognized species generally occur in allopatry (Figure 3), suggesting that geographic isolation is an important process during incipient divergence [30]. It is therefore possible that genetically distinct clades simply reflect geographic differentiation within single species. However, clades within each of *L. guttatus*, *L. elongatus*, and *L. grandis *are often found on a single beach yet remain distinct in the nuclear gene genealogy, which suggests the presence of a reproductive barrier. Also, at least the six genetic clades within *L. elongatus *can be clearly distinguished based on vertebra number, degree of reduction of the pelvic fin sucker, and microhabitat, indicating that the level of *Cyt b *differentiation found in *L. elongatus *(6.8–13.8% uncorrected pairwise difference) represents divergence at the species level. Further genetic, morphological, and ecological studies are therefore likely to reveal additional evidence for the identity of species recovered in this study.

The species-level phylogeny based on combined analysis of six nuclear genes suggested that the genus *Luciogobius *is monophyletic and does not include *Inu koma *and *I. saikaiensis*. Previous inclusion of the *Inu *species in *Luciogobius *is based on the shared absence of the first dorsal fin and scales [13], which likely reflects parallel reduction associated with adaptation to narrow habitats (*e. g*., rocky shores, gravel beaches). The genus *Luciogobius *as treated here clearly differs osteologically from *Inu *and other gobies: the pterygiophore of the first ray of the second dorsal fin is inserted into the caudal vertebra in *Luciogobius*, whereas that of all other gobies is connected to the abdominal column. The two undescribed species of the unplaced genus (Gen. sp. 1 and 2) also share these characteristics with *Luciogobius*, suggesting that they may be the direct sisters of *Luciogobius*. Alternatively, the presence of fleshy maxillary barbels in the two species suggests affinity to species of *Inu *and *Clariger*. Due to lack of statistical support (Figure 4 and Additional file 1), however, the placement of Gen. sp. 1 and 2 remain inconclusive. Considering these circumstances, we tentatively treated these species as *incertae sedis*.

### Evolution of the interstitial habit

**Figure 6:**
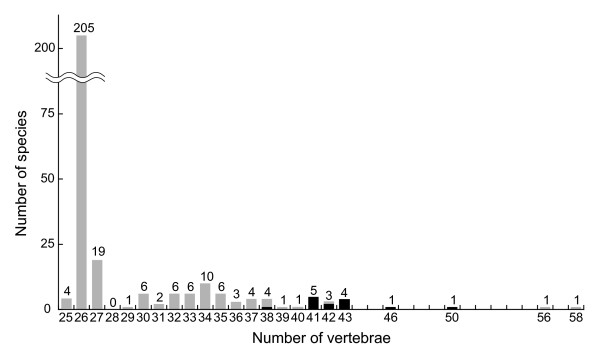
**Frequency distribution of vertebra number among 294 species of Gobiidae occurring in Japan**. Data are based on Akihito *et al*. (1984) and this study. Following the phylogenetic results of Thacker (2003), Gunnellichthyidae *sensu *Akihito *et al*. (1984) is included in Gobiidae. The genetically differentiated taxa uncovered by the *Cyt b *phylogeny are scored individually in the histogram. Interstitial species are indicated by black bars.

The nuclear gene phylogeny clearly suggested that the interstitial species belong to two separate clades (Figure 4). Based on the obtained phylogenetic pattern, the interstitial habit evolved either twice independently in the common ancestors of these two clades, or once in the common ancestor of *Luciogobius *and was lost twice independently in *L. pallidus *and *L. guttatus*. Although there is no rationale for choosing between these alternative hypotheses, in either case, the analysis of independent contrasts indicates that transitions to interstitial habits are strongly correlated with increases in vertebra number. Since an increase in vertebra number results in finer segmentation of the vertebral column and hence improved flexibility of the body, the evolution of an elongated body with a highly segmented vertebral column has likely been one of the key innovations during adaptation to the dynamic gravel environment. The ground vertebra number of gobiid fishes is 26, which is fixed across more than 70% of all goby species occurring in Japan [12] (Figure 6). However, interstitial *Luciogobius *species have far more vertebrae than this number, despite being among the smallest of gobiid fishes (ranging in size from 2 cm in *L. adapel *to 7 cm in *L. grandis*). Gobies with the highest numbers of vertebrae (58 and 56) are medium sized fishes (6–9 cm) of the genus *Gunnellichthys *[12]; therefore, the vertebral column of interstitial *Luciogobius *are among the most finely segmented of all gobiid fishes.

### Diversification of *Luciogobius *in the Japanese Arc

The Japanese Arc is located on compressional margins of four tectonic plates (*i. e*., Okhotsk, Eurasia, Philippine, and Pacific Plates), and this has caused active orogenesis that started ca. 15 Ma in the Middle Miocene [20-22]. The resultant steep mountain ranges and abundant rainfall provided by monsoons have brought a rich supply of terrigenous rocks to the sea via rivers. The steep, strongly indented nature of the Japanese seacoasts and active stirring by waves have also facilitated the accumulation of well-sorted rocks and gravels along the coast [4]. These processes are the major cause of the present-day excessive abundance of gravel beaches in Japan. Importantly in the tropics, where gobies are most diverse, many seacoasts are fringed by coral reef, which diminishes strong wave action and prevents the development of well-sorted gravel beaches.

Considering the uniqueness of Japanese seacoasts, the high species diversity and largely restricted distribution of interstitial *Luciogobius *in the Japanese Arc are mostly feasibly interpreted as the result of *in situ *diversification within the archipelago. In fact, our estimation of the origin of the interstitial species (6–12 Ma; Figure 4, Additional files 3 and 4) is consistent with the initial formation of Japanese mountain ranges being followed by colonization by *Luciogobius *and subsequent diversification. Furthermore, our results suggest that different species of interstitial *Luciogobius *prefer gravel sediments with largely different gravel sizes (Figure 5). This indicates that the granulometric complexity of gravel beaches has provided an opportunity for niche partitioning among major clades of *Luciogobius*, which was probably an important process during the initial stages of their diversification. Although closely related *Luciogobius *species are similar in morphology and may occupy interstitial habitats with similar granulometric parameters, their microhabitats differ with respect to wave action, tidal level, and the presence or absence of boulders, rocks, bedrock, and the inflow of infiltrated fresh water.

### Adaptive radiation of *Luciogobius*

Adaptive radiation is the evolution of ecological and phenotypic diversity within a rapidly diversifying lineage. Schluter [31] proposed four criteria for detecting an adaptive radiation: (1) recent common ancestry, (2) phenotype-environment correlation, (3) trait utility, and (4) rapid speciation. Even the widely acknowledged examples of adaptive radiation rarely fulfil or explicitly test these criteria [31]; thus, it is beyond the scope of the present study to fully substantiate the four criteria. However, available evidence suggests that the present organismal and phenotypic diversity of *Luciogobius *is likely the result of an adaptive radiation in the interstitial habitats of gravel beaches.

Recent common ancestry is clearly the case of interstitial *Luciogobius *species. Although the interstitial species are not monophyletic, they are all descended from a recent common ancestor, which colonized gravel beaches and evolved to utilize various interstitial environments. We found that gravel sediments with different granulometric properties are occupied by different species of interstitial *Luciogobius *(Figure 5). This habitat preference is associated with differences in body size and vertebra number (phenotype-environment correlation), because species having more finely-segmented body tend to inhabit small, well-sorted gravels. For example, *L. elongatus*, *L. adapel*, and *L*. sp., which have the smallest body sizes and highest vertebra numbers of all *Luciogobius *species, inhabit sediments with the most finely sorted gravels, whereas *L. grandis *having larger body and fewer vertebrae prefer coarse gravels (Figure 5). Whether body size and vertebra number affect fishes' performance in different interstitial habitats (trait utility) is not straightforward to assess. Nevertheless, it is likely that fine morphological match to the gravel environment promotes fishes' ability to burrow into the sediment, escape from predators, and/or capture prey in wave-disturbed gravel beaches. Finally, recent ancestry (< 12 Ma) and a remarkable diversity of *Luciogobius *species found within a small geographic area (the Japanese Arc and surrounding regions) are consistent with rapid speciation in *Luciogobius*. Indeed, the number of species in *Luciogobius *far exceeds those found in any other related marine genera (18 in *Luciogobius *vs. five in the next largest genus *Clariger*). Although further study is clearly necessary, the above observations suggest that *Luciogobius *provides a valuable, promising new example of adaptive radiation that took place in a habitat previously considered inaccessible to vertebrates.

## Conclusion

Overall, our findings illuminate the significance of gravel beaches as previously unappreciated theaters of marine fish radiation. *Luciogobius *fishes have adapted to this dynamic environment with elongated bodies and finely segmented vertebral columns, which allowed them to access new niches and radiate into various microhabitats within the gravel beach. Interstitial habitats of gravel beaches are not utilized by any other vertebrate, which has likely been important for these gobies to flourish in such a unique habitat. It is likely that adaptation to interstitial habitats has provided preconditions for the colonization of infiltrating underground water, anchialine caves, or estuarine mudshrimp burrows, which further fuelled the diversification of this unique fish clade. Therefore, the genus *Luciogobius *offers a valuable future model system for studying vertebrate adaptation to interstitial life and adaptive radiation by fishes in an extraordinary habitat.

## Methods

### (a) Sampling

Gobies were collected from various coastal habitats along the Japanese seacoast (Figure 2 and Additional file 6). We sampled seven of the 11 described *Luciogobius *species and one putative undescribed species that does not match any of the known species morphologically (Table 1). *Inu koma *and *I. saikaiensis *are included in *Luciogobius *in most recent classifications [e.g. [13]], but they are treated as a separate genus, which concurs with our phylogenetic results. We also sampled 11 additional species representing eight related genera and two undescribed taxa that are probably related to *Luciogobius*. Among the *Luciogobius *species, four taxa are strictly interstitial and occur only in the intertidal zones of well-sorted gravel beaches [16-19]. Another interstitial species, *L. adapel*, lives in gravel beds at depths of 20~40 m, and has completely lost the first and second dorsal, anal, and pelvic fins [11]. The remaining species are found under rocks in estuary sand beds (*L. guttatus *and *L. dormitoris*) or in underground water in gravel estuaries (*L. pallidus *and *L. albus*) [10,32-34]. *Luciogobius albus*, the only known cavefish in Japan, has been collected only from a few anchialine caves [33], while *L. pallidus *is sometimes found within mudshrimp burrows [35]. We were unable to sample *L. albus*, *L. dormitoris*, *L. brevipterus*, or *L. matellii*; the last three species are known only from the type collections [10,36,37]. A full list of sampling localities is provided in Additional file 6.

Gobies were collected using either a hand net or by scooping gravel sediments with a shovel and placing them into a net to sort the fish. *Luciogobius adapel *was collected using a bottom sampler at depths of 20 m. For each goby, a small fragment of muscle tissue was removed and kept in absolute ethanol for subsequent extraction of DNA, and the remaining body was kept in 10% formalin solution for morphological examination.

### (b) Molecular protocols

Genomic DNA was extracted from muscle tissue following a standard phenol-chloroform method. Using the polymerase chain reaction (PCR), we amplified fragments of the *Cyt b *gene using the primers detailed in Additional file 7. PCR conditions for amplifying the *Cyt b *fragments consisted of an initial 5 min at 94°C, followed by 30 cycles of 30 sec at 94°C, 30 sec at 45°C, and 1 min at 72°C, with a final extension at 72°C for 7 min. The PCR reaction conditions for the six nuclear genes were the same as those for *Cyt b*, except for the annealing temperatures, which are listed along with the primer sequences in Additional file 7. The PCR products were purified using NucleoSpin Extract (Macherey-Nagel, Düren, Germany). Both strands were sequenced using the ABI Prism dye terminator cycle sequencing ready reaction kit (Perkin-Elmer, Foster City, CA), and an ABI 3100 sequencer. Obtained sequences have been deposited in the DNA Data Bank of Japan (DDBJ) database [DDBJ: AB503858–AB504232].

### (c) Phylogenetic analysis

The alignment was straightforward and required only a few unambiguous gaps in the *Mll *intron. Analyses were conducted separately for the *Cyt b *gene and the combined nuclear genes, and for each dataset, phylogenetic trees were obtained using maximum parsimony (MP), maximum likelihood (ML), and Bayesian methods. Because saturation of base substitution was suspected in the *Cyt b *data, we partitioned the data by codon positions and plotted transitions against transversions to detect potential saturation [38] using PAUP* version 4.0b10 [39]. This analysis suggested that the third codon position of the *Cyt b *gene suffered from substitution saturation (Additional file 8). However, trees reconstructed with and without the third codon position did not differ significantly in terms of resolution and branch support; thus, we only report results obtained using all positions combined.

Using PAUP*, we performed MP analysis with 100 random addition analyses and tree bisection-reconnection (TBR) branch-swapping and we validated the robustness of the MP trees using bootstrap analysis with 1000 replications. Before the ML analysis, the program Modeltest 3.0 [40] was used to select appropriate models of base substitution and to estimate model parameters. ML analysis of the *Cyt b *dataset proved to be very computer-intensive using the TBR swapping algorithm and so was done using PhyML [41], while that of the combined nuclear dataset was performed using PAUP* with ten random addition analyses and TBR branch swapping. ML bootstrap analyses of both datasets were conducted with PhyML with 100 replications. For the Bayesian analysis, we used the program MrModeltest 2.2 [42] to select an appropriate model of base substitution for each gene partition. Based on the selected models, we performed Bayesian analyses using MrBayes 3.1.2 [43] with the unlink command (nuclear dataset only) to estimate substitution parameters separately for each partition. Analyses consisted of running four simultaneous chains for 2 × 10^6 ^generations, sampling trees every 1000 generations for a total of 2001 trees. We plotted the ln-likelihood of the sampled trees against generation time to identify the region of the analysis in which the parameter estimates were stable. Then, we discarded the burn-in region (trees and parameter estimates obtained before equilibrium; the initial 1001 trees), and the remaining 1000 samples were used to estimate the tree topology, branch lengths, and substitution parameters.

### (d) Analysis of vertebra number evolution

Number of vertebrae was counted for 1–19 representative individuals for each taxa identified from the *Cyt b *phylogeny (Additional file 2). Vertebral counts were taken from radiographs produced with Softex Type C-60 (Softex, Tokyo, Japan) exposed at 20 KVp and 4 mA for 20 seconds. Then, the average vertebra number for each taxon was mapped onto the combined nuclear ML phylogeny, and ancestral state estimates of vertebra number were obtained using the squared change parsimony algorithm as implemented in Mesquite version 1.12 [44]. To test for correlated evolution between interstitial habitat use and vertebra number along cladogenesis, we calculated independent contrasts [23] of each of the variables using the PDAP module of Mesquite [45]. Each species was assigned as either interstitial (1) or non-interstitial (0) as designated in Table 1. The use of discrete character states does not violate the assumption of independent contrasts [46].

### (e) Estimation of divergence time

Combined nuclear gene ML phylogeny was transformed to an ultrametric tree using the penalized-likelihood relaxed clock approach with the program r8s [25]. Since a reliable maximum prior for the age of the root node is not available, we first obtained a linearized tree by fixing the age of the root node at one arbitrary time unit (without any internal constraints) with a smoothing parameter of 10 as inferred by cross validation. This analysis produces an ultrametric tree with fixed relative node heights but without absolute ages. Divergence times on this tree were then calibrated using (1) the split between *Gymnogobius isaza *and *G. urotaenia*, which was previously estimated at 2.3 Ma based on geological calibration [24] and (2) the divergence rate of the *Cyt b *gene. For the later approach, every node on the ultrametric tree obtained by penalized likelihood was used individually as a calibration point to obtain a conservative range of estimates for the MRCA of the interstitial *Luciogobius *species. We used the *Cyt b *divergence rate because it is generally conserved across fish taxa, has been used previously for dating in gobies [47], and clusters at approximately 2.7% per My in gobiid taxa [24]. Using the 2.7% per My substitution rate, the average pairwise ML distance across each node was transformed to absolute age, and this was used individually as a calibration point. We obtained 95% credibility intervals of node estimates by producing 100 bootstrapped datasets using the SeqBoot program in the PHYLIP package [48], estimating branch lengths of the above ML phylogeny under the substitution model used in the ML calculations, and performing divergence date estimates as described above.

In addition to the above two approaches using internal calibration points, we also adjusted the age of the root node so that the overall rate of *Cytb *substitution equals the previously inferred rate of 0.0135 substitutions/site/My, which is half the 2.7% divergence rate [24]. We first calculated branch lengths of the ML topology using the *Cyt b *data with a substitution model and parameters inferred using Modeltest. This tree was linearized by fixing the age of the root node, and substitution rate along each branch calculated, using the program r8s. We iterated the age of the root node until an average substitution rate comparable to 0.0135 substitutions/site/My was found. The inferred root node age was then used to estimate the age of the MRCA of interstitial *Luciogobius *by performing a penalized likelihood analysis on the combined nuclear ML phylogeny.

### (f) Habitat assessment

We obtained median gravel size and sorting index of the sediment for five interstitial species (*L. grandis*, *L. platycephalus*, *L. elongatus*, *L. adapel*, and an undescribed species *Luciogobius *sp.). When we collected the gobies, we also sampled 1–4 litres of sediment from the habitats where the gobies were found using a shovel. Sediment samples were passed through a series of standard sieves with running water, dried separately, and weighed. Then, the median particle size and sorting index were calculated using the equation given in [28], and between-species differences in these indices were tested using one-way analysis of variance (ANOVA).

## Authors' contributions

TY designed the study, carried out the mitochondrial and nuclear gene sequencing, performed the phylogenetic and granulometric analyses and drafted the manuscript. TS carried out the mitochondrial gene sequencing and participated in the phylogenetic analysis. TN carried out the mitochondrial and nuclear gene sequencing and participated in the phylogenetic analysis. AK performed the phylogenetic analysis and drafted the manuscript. MK conceived of the study, and participated in its design and helped to draft the manuscript. All authors read and approved the final manuscript.

## Supplementary Material

Additional file 1 **Maximum likelihood phylogeny based on 4813 bp of the combined *Mll*, *Myh6*, *Ptr*, *Rag1*, *Rag2*, and *Ryr3 *genes**. Numbers above branches indicate maximum parsimony and maximum likelihood bootstrap values, followed by Bayesian posterior probabilities. Click here for file

Additional file 2 **Vertebral count for each taxa identified on the *Cyt b *phylogeny.** Click here for file

Additional file 3 **Estimated ages of the most recent common ancestor of interstitial *Luciogobius *species**. Every node on the ultrametric tree obtained by penalized likelihood was used individually as a calibration point to obtain a conservative range of estimates for the most recent common ancestor of the interstitial *Luciogobius *species. Optimal ages and 95% credibility intervals are plotted against the relative node heights of those used to calibrate the chronogram. The 7.67 Ma estimate obtained based on *Gymnogobius isaza*-*G. urotaenia *split (2.3 Ma) is given by the dotted line, and 95% credibility interval of this estimate is indicated by the shade. Click here for file

Additional file 4 **Optimal age of the root node inferred based on *Cyt b *substitution rate**. The age of the root node (*Eutaeniichthys gilli*-*Typhlogobius californiensis *split) was adjusted using an iterative approach until average substitution rates across branches equaled the known rate of 0.0135 substitutions/site/My [24]. Click here for file

Additional file 5 **Median gravel size and sorting index for five interstitial *Luciogobius *species.** Click here for file

Additional file 6 **List of species examined and locality information.** Click here for file

Additional file 7 **List of primers used in this study.** Click here for file

Additional file 8 **Plots of uncorrected pairwise transition distances against transversion distances in each of the three *Cyt b *codon positions.** Click here for file
