# Supplementary figures and images for: Adaptive radiation of gobies in the interstitial habitats of gravel beaches accompanied by body elongation and excessive vertebral segmentation
Source: BMC Evol Biol. 2009 Jun 28;9:145. doi: 10.1186/1471-2148-9-145 (PMC2709658; doi:10.1186/1471-2148-9-145)

# *Luciogobius*

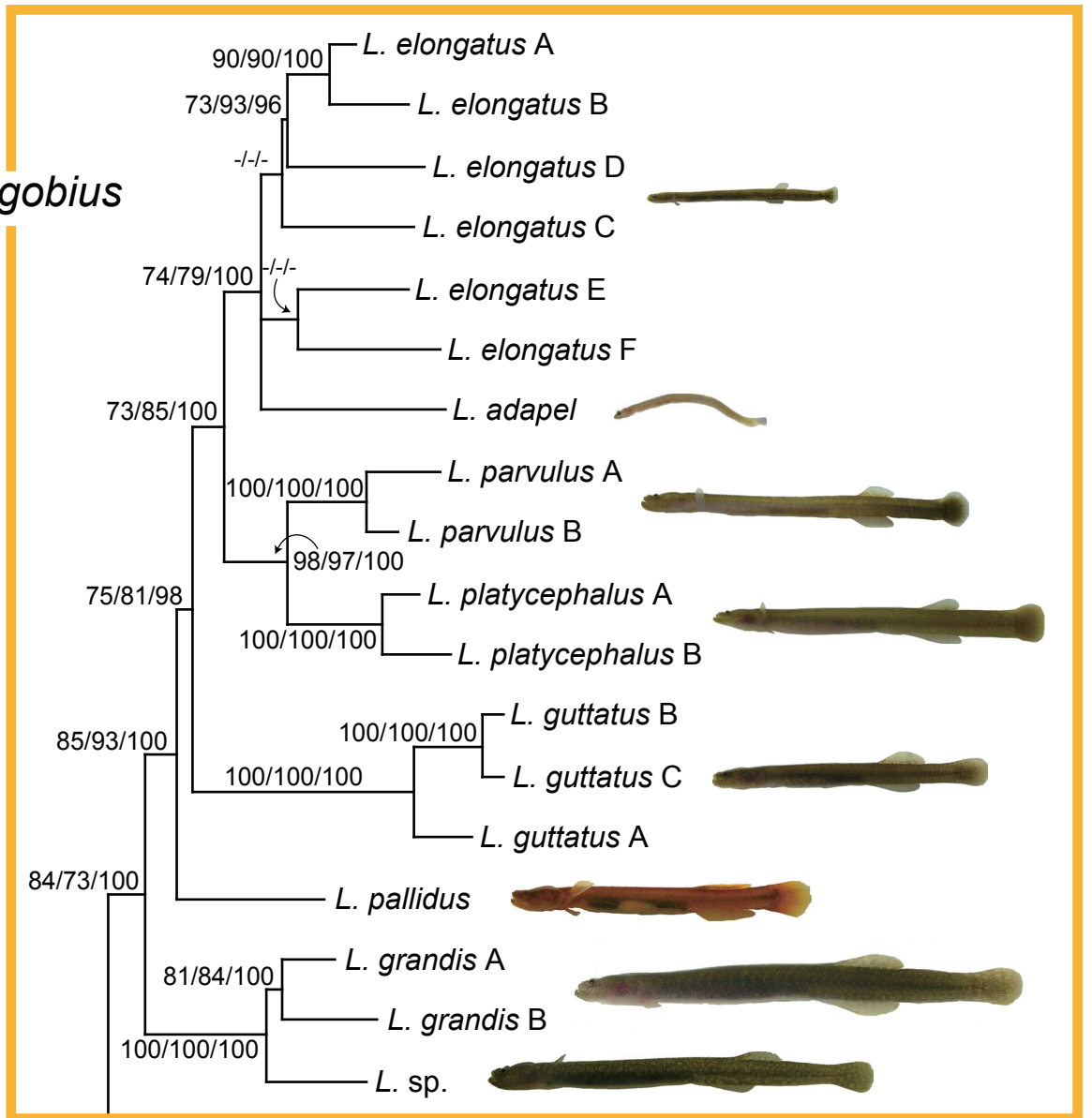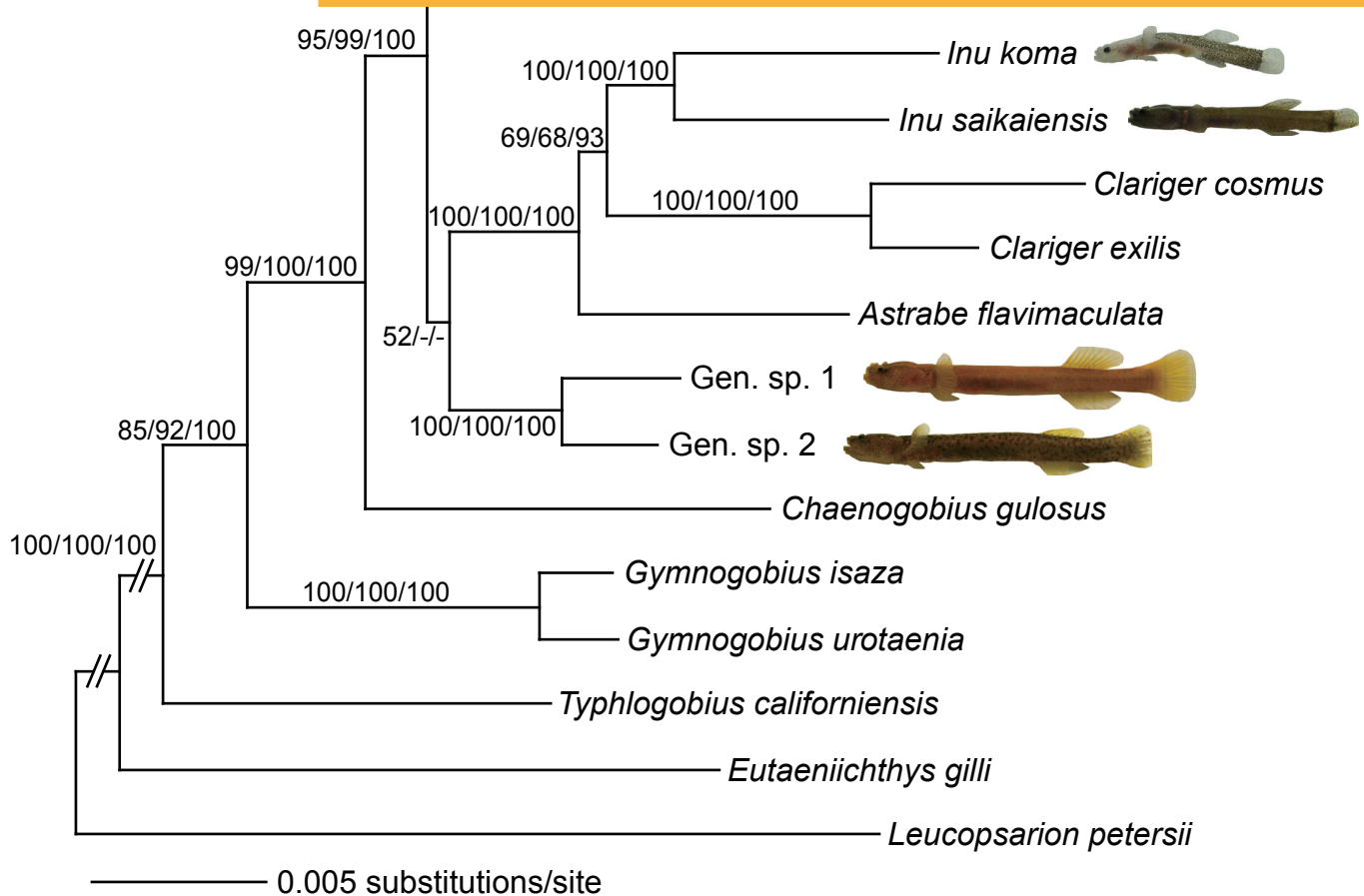

Supplement: Additional file 1 — Maximum likelihood phylogeny based on 4813 bp of the combined Mll, Myh6, Ptr, Rag1, Rag2, and Ryr3 genes. Numbers above branches indicate maximum parsimony and maximum likelihood bootstrap values, followed by Bayesian posterior probabilities. [file 1471-2148-9-145-S1.pdf]

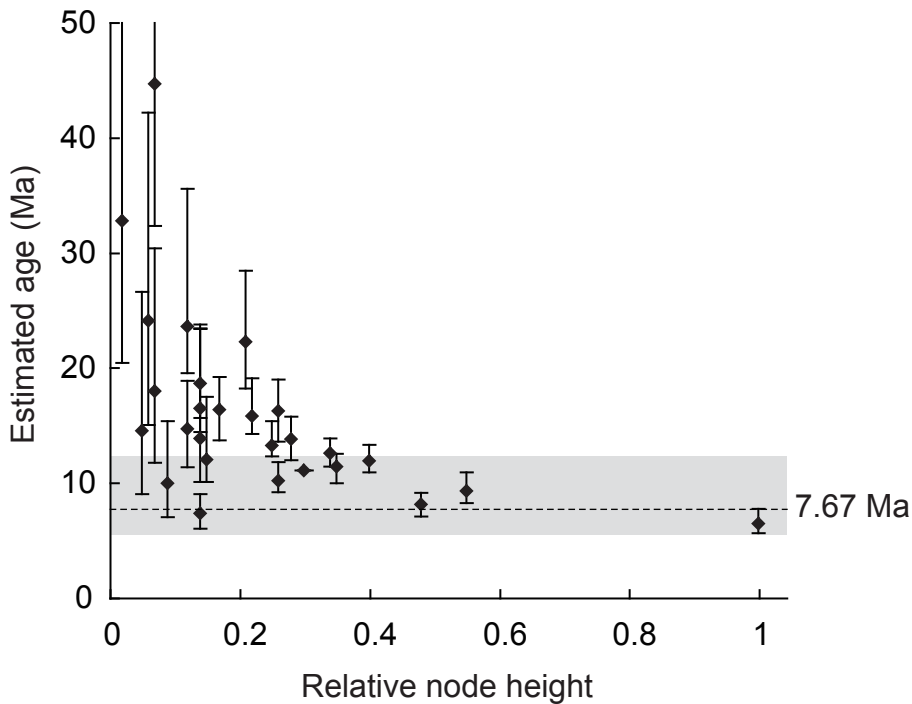

Supplement: Additional file 3 — Estimated ages of the most recent common ancestor of interstitial Luciogobius species. Every node on the ultrametric tree obtained by penalized likelihood was used individually as a calibration point to obtain a conservative range of estimates for the most recent common ancestor of the interstitial Luciogobius species. Optimal ages and 95% credibility intervals are plotted against the relative node heights of those used to calibrate the chronogram. The 7.67 Ma estimate obtained based on Gymnogobius isaza-G. urotaenia split (2.3 Ma) is given by the dotted line, and 95% credibility interval of this estimate is indicated by the shade. [file 1471-2148-9-145-S3.pdf]

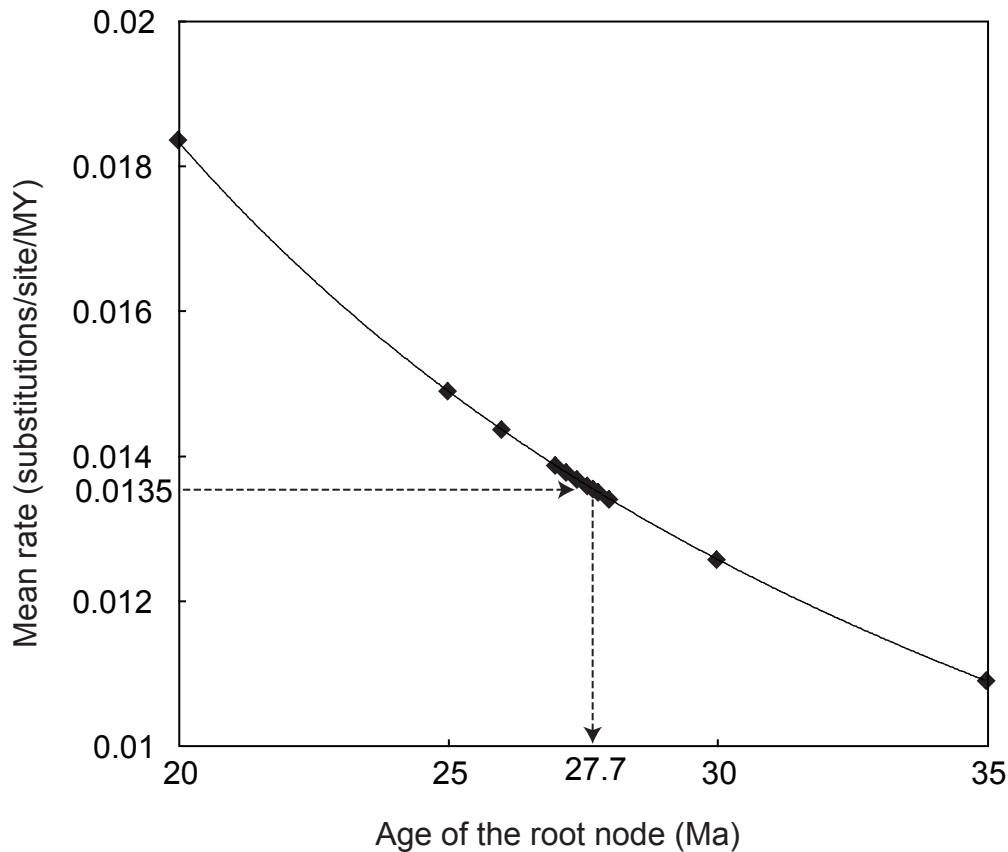

Supplement: Additional file 4 — Optimal age of the root node inferred based on Cyt b substitution rate. The age of the root node (Eutaeniichthys gilli-Typhlogobius californiensis split) was adjusted using an iterative approach until average substitution rates across branches equaled the known rate of 0.0135 substitutions/site/My [24]. [file 1471-2148-9-145-S4.pdf]

## 1st and 2nd codon position

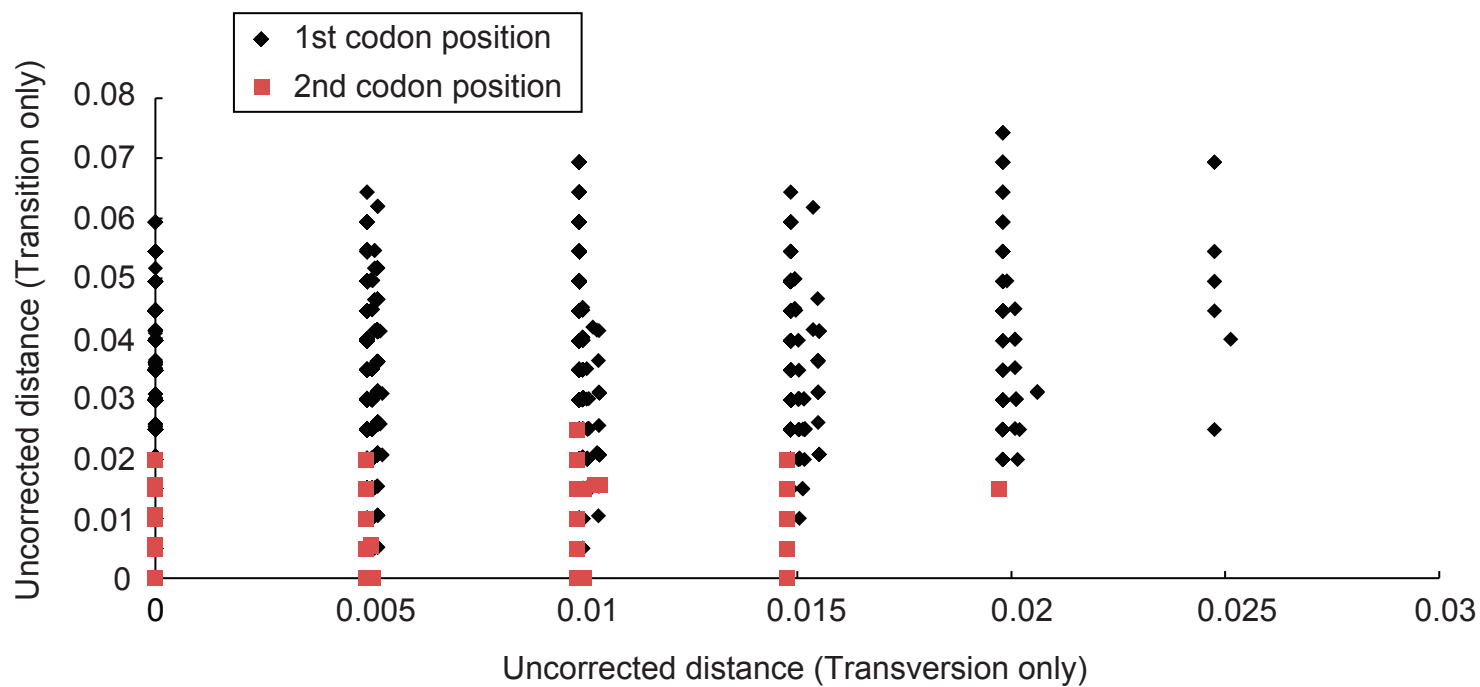

## 3rd codon position

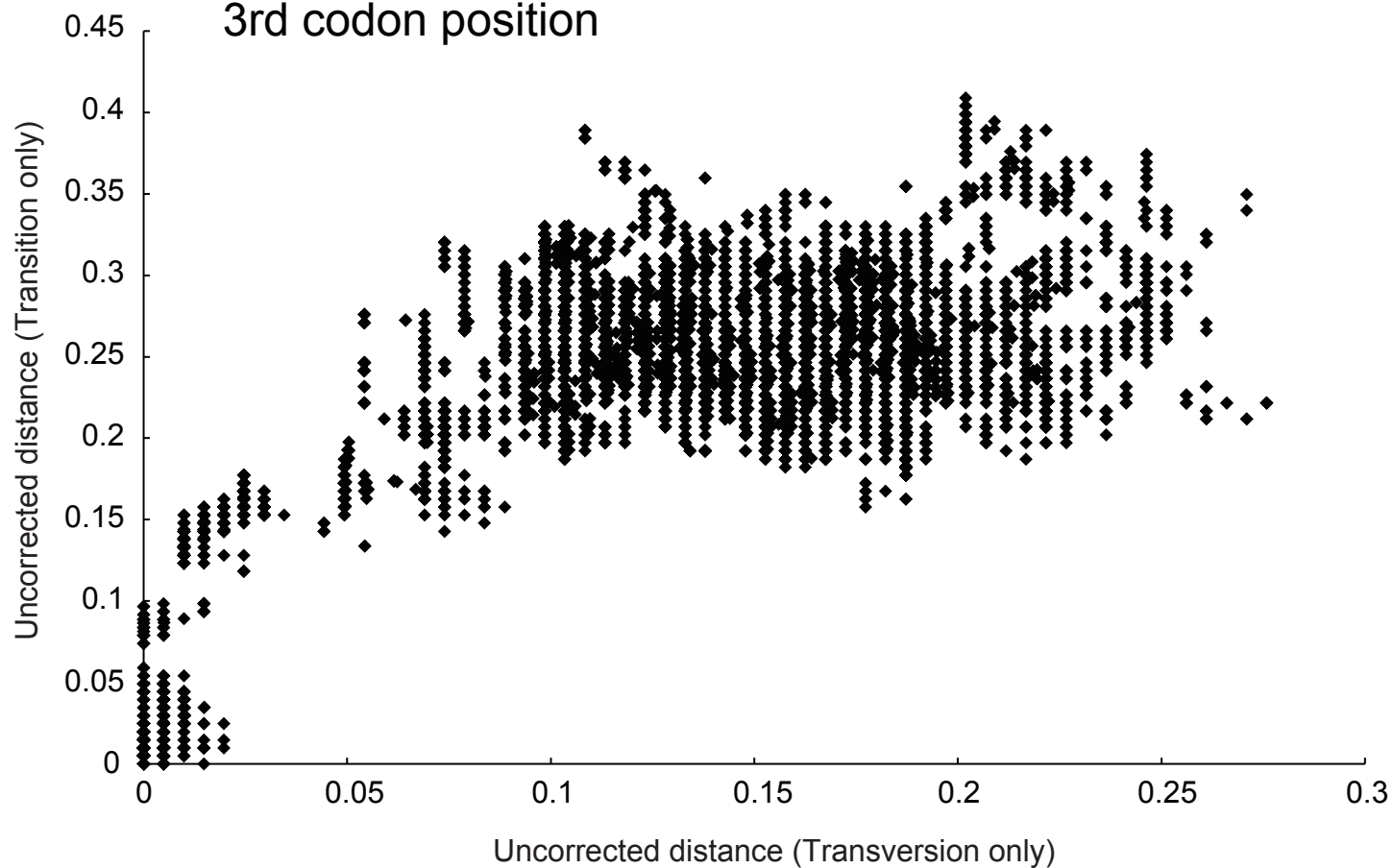

Supplement: Additional file 8 — Plots of uncorrected pairwise transition distances against transversion distances in each of the three Cyt b codon positions. [file 1471-2148-9-145-S8.pdf]
